# Clinical communication skills and professionalism education are required from the beginning of medical training - a point of view of family physicians

**DOI:** 10.1186/s12909-018-1141-2

**Published:** 2018-03-20

**Authors:** Camila Ament Giuliani dos Santos Franco, Renato Soleiman Franco, José Mauro Ceratti Lopes, Milton Severo, Maria Amélia Ferreira

**Affiliations:** 1 School of Medicine (discipline of Family Medicine), Pontifical University of Paraná, Curitiba, Brazil; 2 Department of Medical Education and Simulation, Faculty of Medicine, University of Porto, Alameda Prof. Hernâni Monteiro, 4200-319 Porto, Portugal; 3 School of Medicine (discipline of Introduction to the Medical Practice), Pontifical University of Paraná, Curitiba, Brazil; 4 Department of Public Health, Federal University of Health Sciences of Porto Alegre (UFCSPA), Porto Alegre, Brazil; 5 Community Health Service of the Conceição Hospital Group, R. Sarmento Leite, 245 - Centro Histórico, Porto Alegre, RS 90050-170 Brazil; 6 Department of Epidemiology, Predictive Medicine and Public Health, Faculty of Medicine, University of Porto, Porto, Portugal; 7 Institute of Public Health, University of Porto, Portugal. Alameda Prof. Hernâni Monteiro, 4200-319 Porto, Portugal; 8 Faculty of Medicine, University of Porto, Porto, Portugal

**Keywords:** Communication, Medical education, Professionalism, Primary care, Family physician

## Abstract

**Background:**

The Brazilian undergraduate medical course is six years long. As in other countries, a medical residency is not obligatory to practice as a doctor. In this context, this paper aims to clarify what and when competencies in communication and professionalism should be addressed, shedding light on the role of university, residency and post-residency programmes.

**Methods:**

Brazilian family physicians with diverse levels of medical training answered a questionnaire designed to seek a consensus on the competencies that should be taught (key competencies) and when students should achieve them during their medical training. The data were analysed using descriptive statistics and correlation tests.

**Results:**

A total of seventy-four physicians participated; nearly all participants suggested that the students should achieve communication and professionalism competencies during undergraduate study (twenty out of thirty competencies – 66.7%) or during residency (seven out of thirty competencies – 23.33%). When competencies were analysed in domains, the results were that clinical communication skills and professionalism competencies should be achieved during undergraduate medical education, and interpersonal communication and leadership skills should be reached during postgraduate study.

**Conclusion:**

The authors propose that attainment of clinical communication skills and professionalism competencies should be required for undergraduate students. The foundation for Leadership and Interpersonal Abilities should be particularly formed at an undergraduate level and, furthermore, mastered by immersion in the future workplace and medical responsibilities in residency.

**Electronic supplementary material:**

The online version of this article (10.1186/s12909-018-1141-2) contains supplementary material, which is available to authorized users.

## Background

Better communication competencies and professionalism are crucial for better healthcare outcomes [1, 2]. Effective communication is central to the quality of healthcare [3] because it promotes shared decision-making and improves adherence to therapeutic instructions, to both the patients’ and the physicians’ satisfaction [4–7]. Professionalism is also important and involves competencies that enable doctors to serve the patients’ interests above their own by exercising altruism, accountability, excellence, duty, service, honour, integrity and respect for others [8]. Communication competencies and professionalism have as a common core the building of adequate relationships among patients and their families, the community, and healthcare workers.

The status of teaching communication skills, as stated by Brown (2008), has changed from ‘nice to know’ to ‘need to know’ [9]. Numerous studies conducted in different countries and settings have demonstrated the effectiveness of teaching communication and professionalism during undergraduate [10, 11] and postgraduate medical training [12, 13] for the promotion of good outcomes for patients. Communication skills and professionalism are related to the more influential aspects of healthcare provided by primary care doctors [14] and they are described as being among the fundamental tools for family physicians [4], particularly for the treatment of chronic diseases such as diabetes and hypertension. Undergraduate training in family medicine can promote the improvement of competencies related to communication and professionalism [15], and as the majority of healthcare is initially delivered in the primary care setting, medical education must consider and empower this setting for medical training [16].

Brazilian medical training involves six years of undergraduate courses followed by a residency programme and continued education and training. In Brazil, the residency programme is not obligatory, and after medical school, a physician can work as a doctor without specialisation. Since 2001, communication competencies and professionalism have been defined among outcomes in the Brazilian national guidelines for medical undergraduates [17]. In 2014, the current guideline, *Diretrizes Curriculares Nacionais para o Curso de Graduação em Medicina* (National Curricular Guidelines for the Medicine Course), reinforced communication competencies and professionalism [18]. The teaching of communication competencies and professionalism are now being fostered in Brazilian schools of medicine, where curricula have been structured to include these topics [19].

Family physicians and the primary care setting have become more important in the public health system and in undergraduate and postgraduate medical education in Brazil [18]. Therefore, considering the importance of teaching communication and professionalism to primary care physicians and the importance of the government’s stimuli to include this setting in medical training, the viewpoint of family physicians with regard to medical education has become important for the development of teaching these topics.

A variety of globally accepted documents on medical training have pointed to professionalism and communication competencies as being the competencies that must be achieved [18, 20–28]. Despite being seen as crucial, the teaching, learning and practice of these competencies can be incomplete or even missing among medical students and physicians [29]. Finding consensus on these competencies, and aggregating them into domains or major themes, and then determining the most appropriate time to attain these competencies should help with the design of teaching strategies. Drawing on the importance of family medicine in medical schools and primary care along with the inclusion of the expertise of family physicians in the development of these competencies, this study aims to do the following: 1) define key competencies (KCs) in communication and 2) shed light on when communication and professionalism KCs should be achieved during medical training.

## Methods

### Survey design

The research was performed between January and November 2015. It was developed in three phases: 1) thematic organisation of communication skill competencies and the definition of communication KCs, 2) confirmatory analysis of the themes within communication competencies, and 3) investigation of when family physicians think competencies in professionalism and communication should be achieved. The definition of KCs for professionalism was based on a systematic review by Birden et al. [30], which our research team used to conduct a thematic analysis of relevant papers on professionalism from Birden et al.’s review (the results of which we have already published and presented at conferences [31]). Thus, the first two phases of this study apply to communication and the third phase (when to achieve) is in regard to both communication and professionalism. The participants answered questions about the clustering of the communication competencies, and the professionalism competencies were touched on in part when they answered questions about when the competencies should be achieved. The results, discussion and conclusions of the clustering of competencies are only applied to the communication competencies.

### The thematic organisation of communication competencies

KCs for communication were thematically organised by examining medical training reference documents and reviewing the consensus statements of clinical communication skills. This included a total of nine documents: six medical training guides from the UK, Germany, the United States, Canada, Australia and Brazil; and three documents focused on communication skills, the Calgary–Cambridge Guide, the Kalamazoo Consensus Statement and the European consensus on learning objectives for a core communication curriculum in health care professions [18, 20–28].

Three researchers performed a thematic organisation of these documents using a three-step process. The three researchers each have experience in medical education and one has been involved in medical training and training of trainers in communication and patient-centred medicine, for more than thirty years. Two are family physicians and one is a psychiatrist. In the first step, all competencies and learning outcomes related to communication (‘fragments’) were identified and highlighted. In the second step, the fragments were grouped by content similarity, and descriptive themes were generated without any preconceived determinations on the part of the researchers. These descriptive themes became the KCs, which needed to be representative of the core meanings of the fragments’ contents (the fragments corresponded to the highlighted learning outcomes related to communication in the reference documents). Once these KCs for communication were determined, they were grouped into three domains: Clinical Communication Skills (CCS), closely related to patient care; Interpersonal Communication (IC), closely related to teamwork, multidisciplinary teams and colleagues; and Leadership.

The second phase used Qualtrics™, a web-based survey system. The survey was sent by email to 213 doctors from all regions of Brazil between April and June 2015 (Additional file 1- Survey Questionnaire). The recipients had participated in courses for trainers in family medicine under three types of professional activities: preceptors, faculty members and medical doctors. The questionnaire included three sections: 1) collection of demographic characteristics, professional activities and an estimation of the time frame within which each doctor had achieved their own competencies in communication skills and professionalism; 2) confirmation of the communication themes (KCs) defined by the researchers and 3) their point of view on the optimal time to require attainment of these KCs, i.e. at the undergraduate level, during residency or after residency.

In the second section of the questionnaire, to confirm if the competencies were appropriately represented by the KCs, the fragments were presented to 74 subjects who were asked to choose a KC that best represented the fragment’s meaning. Among the choices was one or more of the KCs as well as others not included in the KCs.

### Statistical analyses

The categorical numerical variables were described using counts (percentages) and means (SD) or medians (25th percentile and 75th percentile). To find the groups of fragments that corresponded to each KC, the participants’ responses were evaluated. To identify the response patterns, we used hierarchical clustering with Manhattan distance. The number of clusters was identified using fusion coefficients.

To find the best time for each KC to be attained, all KCs in each domain were scored from 0 to 100. The Mann–Whitney test and Spearman’s rank correlation were used to determine whether there were significant associations between any KCs and the overall scores. Data were analysed using SPSS, Version 22.0.

## Results

### Characteristics of the sample’s subjects

The 74 (34.6%) participants ranged in age from 27 to 59 years old, with a mean age of 37.9 years (SD = 7.6). They had graduated from medical school 13.6 (mean) years ago (SD = 7.8), between 1980 and 2012, and 51.4% were women. Ninety-three per cent had worked as a family physician for 12.2 (mean) years (SD = 8.0), 91.9% had worked as a preceptor for 5.4 (mean) years (SD = 5.7) and 71.4% had worked as a faculty member for 6.0 years (mean) (SD = 5.6).

Most of the participants (72.9%) had completed residency programmes in family medicine that certified them as family physician specialists and 18.9% were family physician specialists without a residency. Only 8% were not family physician specialists but they had worked in or taught primary healthcare courses. Forty-six per cent had a master’s degree, 5.4% had a doctoral degree and 1.3% had a post-doctorate degree.

### The definition of KCs in communication

The 9 documents examined for KCs contained 88 communication competencies (highlighted fragments) [18, 20–28]. Thirty-one competencies (fragments) were identical or very similar across the documents, leaving fifty-seven competencies. The thematic organisation categorised these 57 fragments into 18 KCs; 11 KCs were conceptually related to CCS, 5 were related to IC and 2 were related to Leadership.

When the participants chose the KCs that best represented a particular competency’s idea and meaning, the answers generated groups of competencies that were associated with the KCs (there was one or more competencies related to each KC). The confirmation of these KCs by the family physicians and the percentage of agreement are shown in Table 1, where frequency means how often the whole group of competencies were related to the KC for CCS. For the competencies in IC (10 competencies) and in Leadership (7 competencies), the aggregation of competencies into groups was not considered because of the small number of items. There were no fragments not represented by any KC.

**Table 1:** The percentage of agreement among Family Physicians on Key Competencies and the competencies represented by each group of Key Competencies

Clinical Communication Skills (Key Competencies)	Group of Competencies (Fragments) (CCS)*											
	1	2	3	4	5	6	7	8	9	10	11	12
Communicate effectively according to given roles	44%	3%	2%	1%	7%	7%	5%	3%	2%	0%	0%	0%
Establish a therapeutic and professional relationship	4%	1%	42%	0%	2%	0%	1%	0%	0%	5%	0%	0%
Build a suitable relationship	3%	49%	29%	6%	7%	6%	1%	2%	0%	0%	0%	0%
Involve the bio-psycho-social context	1%	0%	0%	7%	2%	8%	0%	2%	0%	0%	2%	84%
Understand the perspective of the patient and his or her family	2%	1%	3%	63%	6%	2%	5%	2%	6%	0%	2%	11%
Adapt communication according to the patient and his or her family	18%	5%	3%	3%	1%	71%	8%	2%	2%	0%	3%	0%
Engage patients and families to share in decision-making	3%	27%	9%	10%	11%	0%	10%	0%	0%	5%	63%	0%
Support decision-making based on the needs and interests of the patient	1%	8%	8%	7%	13%	3%	15%	4%	0%	79%	12%	5%
Structure and organize communication/clinical interviews	6%	2%	0%	1%	37%	1%	6%	85%	2%	5%	1%	0%
Inform patients and family adequately	9%	0%	2%	0%	2%	2%	40%	0%	0%	0%	14%	0%
Communicate bad news appropriately	5%	0%	0%	2%	0%	2%	4%	0%	87%	0%	0%	0%
None of the Key Competencies	4%	3%	2%	1%	11%	0%	3%	2%	0%	5%	0%	0%
Group of Competencies (Fragments) (CCS)*	Competency (Fragments)											
1	Communicate effectively with patients, families and the public											
	Communicate effectively in wider roles including health advocacy, teaching, assessing and appraising. Communicate effectively about ethical issues with patients and family											
	Communicate effectively in various roles, for example, as patient advocate, teacher, manager or improvement leader											
	Uses effective and efficient communication and management strategies											
	Demonstrate by listening, sharing and responding, the ability to communicate clearly, sensitively and effectively with patients and their family/careers											
	Communicate clearly, sensitively and effectively with patients, their relatives or other carers, and colleagues from the medical and other professions, by listening, sharing and responding											
	Demonstrate by listening, sharing and responding, the ability to communicate clearly, sensitively and effectively with patients and their family/careers											
2	Building relationship (Using appropriate non-verbal behaviour, Developing rapport and Involving the patient)											
	Shaping of relationship: involves the patient in the interaction using a patient-centered approach											
	Recognizes the patient as a partner in shaping a relationship											
3	Involves the patient in the interaction to establish a therapeutic relationship using a patient-centered approach.											
	Relates to the patient respectfully including ensuring confidentiality, privacy and autonomy...											
	Establish professional therapeutic relationships with patients and their families											
	Create and sustain a therapeutic, ethical relationships with patients											
4	Gathering information (Exploration of patient’s problems and Additional skills for understanding the patient’s perspective)											
	Encourage the patient to express own ideas, concerns, expectations and feelings and accepts legitimacy of patients views and feeling											
	Understand the Patient’s Perspective											
5	Gather information											
	Information: effectively collects the relevant information for the reasoning and decision-making process											
	Effectively collects relevant information for reasoning and decision making											
	Initiating the session (Establishing initial rapport and Identifying the reason(s) for the consultation)											
	Open the Discussion											
6	Communication in the doctor–patient relationship: orients her communication behaviour along the actual concerns and the personality of the patient											
	Social behaviour and communication: adapts her social behaviour and communication to different social contexts and communication partners.											
	Adapts own communication to the level of understanding and language of the patient, uses techniques aproppriates for this											
7	Explanation and planning (Providing the correct amount and type of information, Aiding accurate recall and understanding...)											
	Information: effectively communicates the relevant information for the reasoning and decision-making process											
	Effectively communicates relevant information for reasoning and decision making. Gives information to the patient in a timely, comprehensive and meaningful manner											
	Elicit and synthesize accurate and relevant information, incorporating the perspectives of patients and their families											
8	Shapes a conversation from beginning to end with regard to structure (e.g. introduction, initiating the conversation, gathering and giving information, planning, closing interview, setting up next meeting; time management)											
	Providing structure (Making organization overt And Attending to flow)											
9	Communicate appropriately with difficult or violent patients; people with mental illness and vulnerable patients//Communicate appropriately in difficult circumstances, such as when breaking bad news, and when discussing sensitive issues, such as alcohol consumption, smoking or obesity											
	Recognizes difficult situations and communication challenges and deals with them sensitively and constructively											
10	;...developing plans that reflect the patient’s health care needs and goals											
11	Involve patients in decision-making and planning their treatment, including communicating risk and benefits of management options.											
	Engage patients and their families...											
	Share information											
	Explanation and planning (Achieving a shared understanding: incorporating the patient’s perspective and Planning: shared decision making)											
	Share health care information and plans with patients and their families											
12	Elicits and explores the content of the patient’s bio-psycho-social history.											
Interpersonal Communication (Key Competencies)	Number (IC)											
	1	2	3	4	5	6	7	8	9	10		
Work effectively in multidisciplinary team, adapting to the particularities of each team and given roles	72%	30%	87%	55%	25%	9%	12%	0%	0%	0%		
Perform consulting, helping colleagues, other professionals, and the healthcare system	11%	26%	0%	0%	39%	82%	6%	5%	0%	0%		
Communicate effectively to promote understanding and resolve conflicts, aiming to ensure the success of teamwork	11%	19%	13%	30%	6%	0%	65%	11%	82%	18%		
Perform teamwork, aiming to ensure patient safety	6%	7%	0%	10%	9%	0%	6%	79%	0%	5%		
Communicate about ethical issues with other health professionals	0%	4%	0%	0%	0%	0%	0%	0%	18%	77%		
None of the Key Competencies	0%	15%	0%	5%	19%	9%	11%	5%	0%	0%		
Number (IC)	Competency (Fragments)											
1	Work effectively with physicians and other colleagues in the health care professions											
2	Communicate effectively with physicians, other health professionals and health-related agencies											
3	Team building and working in a team: adapts her behaviour to different phases of team building and efficiently shapes her working style to contribute to a successful team											
4	Shows ability to communicate effectively in multi-professional teams											
5	Contribute to the improvement of health care delivery in teams, organizations, and systems											
6	Act in a consultative role to other physicians, health-related agencies and policy-makers											
7	Work with physicians and other colleagues in the healthcare professions to promote understanding, manage differences, and resolve conflicts											
8	Demonstrate by listening, sharing and responding, the ability to communicate clearly, sensitively and effectively with doctors and other health professionals.											
9	Hand over the care of a patient to another health care professional to facilitate continuity of safe patient care											
10	Communicate effectively about ethical issues with health care professionals.											
Leadership (Key Competencies)	Number (Leadership)											
	1		2		3	4		5	6		7	
Demonstrate basic leadership skills	94%		82%		80%	73%		100%	24%		14%	
Engage in the management of human and health care resources	0%		9%		5%	20%		0%	76%		81%	
None of the Key Competencies	6%		9%		15%	7%		0%	0%		5%	
Number (Leadership)	Competency (Fragments)											
1	Demonstrate leadership in professional practice											
2	Work with other care providers as a team leader or member											
3	Describe the principles and practice of leadership in health care.											
4	Leadership: shows basic competencies in leadership skills and supports the development and maintenance of the teamwork with her behavior											
5	Shows basic competencies in leadership skills											
6	Engage in the stewardship of health care resources.											
7	Manage career planning, finances, and health human resources in a practice											

The definition of KCs for professionalism was based on the articles referred to in the systematic review by Birden et al. [30], as relevant to this theme. The articles were read in full and the competencies were extracted from the texts using thematic analysis [31]. Altruism; accountability; humanistic values; ethics; social commitment; commitment to excellence and advancement of knowledge; reflective thinking; dealing effectively with uncertainty and changes; collaboration and teamwork; and expert knowledge were related to the competencies of professionalism. The competencies of collaboration and teamwork were cited in two of the nine papers reviewed for professionalism, but almost all of the documents related to communication included collaboration and teamwork in the fields of interpersonal communication or leadership. Therefore, collaboration and teamwork were presented in the communication competencies, namely in the KCs for IC and Leadership. Consequently, there were 9 professionalism themes represented by 12 KCs (Table 2).

**Table 2:** When students should achieve professionalism and communication competencies

Competency	Subdomain Competencies	Under graduate %	FM Residency %	After Residency %	p*
Communicate effectively according to given roles	CCS	50.0	42.1	7.9	0.003
Establish a therapeutic and professional relationship	CCS	35.1	54.1	10.8	0.005
Build a suitable relationship	CCS	60.5	34.2	5.3	< 0.001
Involve the bio-psycho-social context	CCS	48.9	44.4	6.7	< 0.001
Understand the perspective of the patient and his or her family	CCS	70.6	29.4	0.0	0.016
Adapt communication according to the patient and his or her family	CCS	73.8	21.4	4.8	< 0.001
Engage patients and families to share in decision-making	CCS	77.8	22.2	0.0	< 0.001
Support decision-making based on the needs and interests of the patient	CCS	71.8	23.1	5.1	< 0.001
Structure and organize communication/clinical interviews	CCS	59.5	38.1	2.4	< 0.001
Communicate bad news appropriately	CCS	17.1	70.7	12.2	< 0.001
Inform patients and family adequately	CCS	52.6	42.1	5.3	< 0.001
Work effectively in multidisciplinary team, adapting to the particularities of each team and given roles	IC	19.4	69.4	11.1	< 0.001
Perform consulting, helping colleagues, other professionals, and the healthcare system	IC	52.4	42.9	4.8	< 0.001
Communicate effectively to promote understanding and resolve conflicts, aiming to ensure the success of teamwork	IC	16.3	53.5	30.2	0.010
Perform teamwork, aiming to ensure patient safety	IC	19.5	70.7	9.8	< 0.001
Communicate about ethical issues with other health professionals	IC	22.2	61.1	16.7	0.002
Demonstrate basic leadership skills	Leadership	41.7	47.2	11.1	0.017
Engage in the management of human and health care resources	Leadership	7.0	37.2	55.8	< 0.001
Know and apply ethics, acting with honesty and respecting ethical and moral values	Professionalism	90.5	9.5	0.0	< 0.001
Act with interest and dedication	Professionalism	100.0	0.0	0.0	< 0.001
Prioritize patient’s/family’s/community’s interests above one’s own	Professionalism	64.4	26.7	8.9	< 0.001
Recognize one’s limits and know when to request support	Professionalism	68.2	31.8	0.0	0.016
Be responsible and careful in one’s actions	Professionalism	69.2	30.8	0.0	0.016
Attempt to promote patient and/or family safety	Professionalism	89.5	7.9	2.6	< 0.001
Act according to the highest standards of excellence and know where to seek knowledge	Professionalism	41.7	36.1	22.2	0.338
Be empathic and respectful, valuing the feelings and wishes of colleagues, patients, teachers, and other professionals	Professionalism	91.9	5.4	2.7	< 0.001
Consider the beliefs, needs, and views of patients/families	Professionalism	81.8	15.9	2.3	< 0.001
Reflect and have good critical skills	Professionalism	65.9	26.8	7.3	< 0.001
Deal with uncertainty appropriately, adapting to different situations and contexts	Professionalism	32.4	54.1	13.5	0.010
Recognize and nurture their own physical and mental health	Professionalism	87.1	9.7	3.2	< 0.001

*p* value < 0.05 was considered statistically significant

### When students should achieve professionalism and communication competencies

Table 2 shows the participants’ answers regarding when students should achieve each KC. Of the 30 KCs (18 for communication and 12 for professionalism), the subjects reported that 20 (66.7%) of the KCs should be achieved during undergraduate medical education, 7 (23.3%) during residency, 1 (3.3%) after residency, 1 (3.3%) during residency or undergraduate education and 1 (3.33%) during any of these periods.

When the KCs were analysed by the domains (three domains each for communication, CCS, IC and Leadership and one domain for Professionalism), the results showed that the participants believed KCs for CCS and Professionalism should be achieved during undergraduate education, and KCs for IC and Leadership should be achieved during postgraduate study (residency and post-residency) (see Table 2).

The stage of medical training at which the participants reported they had reached professionalism and communication competencies themselves correlated significantly with when they suggested students should achieve these competencies. Subjects who assumed that their professionalism or communication competencies had been achieved in the early stages of their medical training agreed that these competencies should be achieved sooner. Participants who had completed a family medicine residency believed that KCs for CCS should be achieved later. The longer a participant had spent working as a preceptor, the more they agreed that the achievement of KCs in IC should occur later (Table 3).

**Table 3:** Factors associated with the belief that competencies should be developed later or sooner

		Clinical communication skills		Interpersonal communication		Leadership		Professionalism	
	N (%)	Median (P25-P75)	*p	Median (P25-P75)	*p	Median (P25-P75)	*p	Median (P25-P75)	*p
Total	74 (100)	21.43 (8.33–40.00)		50.00 (25.00–50.00)		50.00 (31.25–100.00)		12.50 (0.00–21.43)	
Gender									
Female	38 (51.4)	18.33 (7.44–33.33)	0.267	50.00 (25.00–50.00)	0.647	50.00 (25.00–100.00)	0.475	10.00 (0.00–21.43)	0.617
Male	36 (48.6)	23.21 (8.33–41.67)		50.00 (25.00–50.00)		50.00 (50.00–100.00)		14.29 (0.00–22.32)	
Academic degree									
Graduate	35 (47.3)	20.00 (0.00–38.75)	0.378	50.00 (25.00–50.00)	0.482	50.00 (31.25–100.00)	0.942	10.00 (0.00–20.71)	0.341
Postgraduate	39 (52.7)	21.43 (10.56–40.00)		50.00 (27.08–50.00)		50.00 (43.75–100.00)		12.50 (6.70–23.21)	
Has family medicine residency									
Yes	54 (73)	23.21 (10.28–40.00)	0.029	50.00 (25.00–50.00)	0.969	50.00 (50.00–100.00)	0.297	12.50 (0.00–21.43)	0.729
No	20 (27)	7.74 (0.00–28.47)		43.75 (25.22–53.12)		50.00 (25.00–100.00)		10.00 (0.00–23.02)	
Developed communication competence during undergraduate period?									
Yes	9 (12.2)	0.00 (0.00–41.67)	0.001	12.50 (0.00–37.50)	0.009	0.00 (0.00–37.50)	0.021	0.00 (0.00–12.50)	0.112
No	65 (87.8)	21.43 (0.00–8.33)		50.00 (33.33–50.00)		50.00 (50.00–100.00)		12.50 (0.00–25.00)	
Developed communication competence during family medicine residency?									
Yes	40 (54.1)	25.00 (10.28–40.00)	0.589	42.59 (25.00–50.00)	0.489	50.00 (37.50–100.00)	0.814	11.25 (0.00–22.32)	0.834
No	34 (45.9)	20.00 (8.04–35.00)		46.31 (25.00–50.00)		50.00 (37.50–100.00)		12.50 (0.00–21.43)	
Developed communication competence post-residency?									
Yes	30 (40.5)	29.29 (12.50–41.67)	0.102	50.00 (40.62–50.00)	0.038	50.00 (50.00–100.00)	0.398	13.39 (8.33–24.11)	0.405
No	44 (59.5)	20.00 (0.00–33.33)		33.33 (25.00–50.00)		50.00 (25.00–100.00)		10.00 (0.00–21.43)	
Developed professionalism during undergraduate period?									
Yes	29 (39.2)	10.00 (0.00–21.43	0.002	33.33 (18.12–50.00)	0.007	50.00 (25.00–100.00)	0.723	8.33 (0.00–14.29)	0.009
No	45 (60.8)	30.00 (14.29–41.67)		50.00 (33.33–56.25)		50.00 (50.00–100.00)		14.29 (6.25–28.57)	
Developed professionalism during family medicine residency?									
Yes	40 (54.1)	18.33 (8.33–34.72)	0.442	50.00 (25.00–50.00)	0.830	50.00 (25.00–100.00)	0.448	12.50 (0.00–21.43)	0.838
No	34 (45.9)	21.43 (7.44–41.25)		50.00 (25.00–50.00)		50.00 (50.00–100.00)		10.00 (0.00–24.11)	
Developed professionalism post-residency?									
Yes	25 (33.8)	35.71 (16.67–50.00)	0.011	50.00 (37.50–75.00)	0.016	50.00 (50.00–100.00)	0.443	14.29 (0.00–33.33)	0.087
No	49 (66.2)	16.67 (0.00–30.00)		37.50 (25.00–50.00)		50.00 (25.00–100.00)		10.00 (0.00–21.43)	

*p* value < 0.05 was considered statistically significant

There were no statistical differences with respect to when the domains of the competencies should be reached when considering the participants’ academic degree, gender, age, number of years working as a doctor or number of years working as a faculty member (Table 4).

**Table 4:** The association between the number of years working and the later development of competency

	Clinical communication skills	Interpersonal communication	Leadership	Professionalism
	r (*p*-value)	r (*p*-value)	r (*p*-value)	r (*p*-value)
Years as faculty	−0.212 (0.139)	−0.200 (0.172)	−0.286 (0.073)	−0.169 (0.242)
Years as preceptor	0.114 (0.356)	0.263 (0.033)	−0.032 (0.821)	0.053 (0.666)
Years as medical doctor	−0.011 (0.930)	−0.034 (0.782)	−0.084 (0.543)	−0.111 (0.362)
Age	−0.012 (0.922)	0.086 (0.470)	− 0.169 (0.204)	0.069 (0.557)

## Discussion

The 18 communication KCs appeared to be widely acknowledged by family physicians as representative of the 57 competencies. The physicians thought that the KCs for CCS and Professionalism should be achieved during undergraduate medical education and KCs for IC and Leadership skills should be reached during postgraduate study (residency or after).

### The KCs for communication

The very low selection rate for the option ‘None of the Key Competencies (KCs)’ and the aggregation of competencies suggested that the KCs were representative of all competencies. The range of agreement about the grouping of the KC for CCS ranged from 37% to 87%. Even for the response with the least agreement, ‘KC – Structure and organise communication/clinical interviews’, (37%), the selected KC was almost three times more likely to be related than the second-most related competency, which indicated that the KCs were adequately confirmed by the participants. Finding consensus should help in defining learning outcomes for the teaching of these competencies [25, 32]. Therefore, the identification of KCs could facilitate the development of programmes and learning objectives in this field, but it is necessary to conduct more research to assess the applicability and feasibility of teaching these competencies.

### The achievement of professionalism and CCS competencies

KCs for both CCS and Professionalism were cited as competencies required by the end of undergraduate medical education, while an appropriate development of IC and Leadership should be attained during postgraduate study.

Within KCs for Professionalism, ethics, patient safety and humanistic values were highlighted, and more than 80% of the participants indicated that these should be achieved during the undergraduate level. Professionalism is closely linked with humanistic values, such as altruism and accountability, which encourages medical students to understand their responsibilities to their patients and their families, and society [33] and plays an important role in the practice of medicine, according to faculty and patients [34].

Reflection, critical thinking and accountability were also cited as needing to be achieved during the undergraduate level, but to a lesser extent. A doctor’s capacity to adapt to a given context when dealing with uncertainty was the only KC clearly related to postgraduate study (residency). The Draft CanMEDS 2015 Milestones Guide [26] points out the importance of recognising uncertainty during the undergraduate period but indicated that learning to adapt to and ‘deal with’ uncertainty was best accomplished during residency. The development of this kind of competency requires sustained and diverse training, during which, students learn how to deal with the particularities of individual patients and their families, and team members [35].

Thus, the findings suggested a progression in the attainment of Professionalism, starting with the learning of the values of respect and ethics and the promotion of safety in medical care and then enhancing the practice to reach higher levels of skill with regard to reflection, critical thinking and accountability. Considering that none of these elements develops in a linear fashion, they have to be fostered in medical students starting early but the requirement of achieving these skills must be intensified in clinical practice.

Among the KCs for CCS, almost all, related to the patient-centred interview, were designated as important to be attained during undergraduate medical education. This reinforces the importance of teaching these skills since aspects, such as communication structure and patient orientation [36, 37], require specific training in order to achieve the best results in practice [38]. The CCS skills needed during more difficult and specific situations, such as breaking bad news, were proposed as needing to be adequately achieved during residency despite studies showing the effectiveness of teaching this skill during undergraduate and postgraduate study [39]. Frequently, newly graduated physicians must handle these kinds of situations. Therefore, despite the fact that this skill should be attained in residency, undergraduate study must provide a strong foundation for its development [40, 41].

The subjects pointed to during residency or even later in medical training as the ideal time for the attainment of Leadership and IC KCs. These competencies involve many types of clinical contexts, including the need to be able to work in a multidisciplinary team, perform consultations, resolve conflict in order to ensure patient safety, exercise basic leadership skills and engage in the management of human and healthcare resources. Therefore, a profound immersion in the responsibilities of the workplace environment is necessary during postgraduate training [42]. The achievement of significant milestones might build the principles of professional IC and Leadership, as happens with teamwork in student–student and/or student–faculty work [26, 43]. It is important for medical schools to prioritise curricula by first bolstering and evaluating certain competencies and then ensuring a foundation upon which to later build and achieve others.

Defining when to achieve a KC is an important step, but the growth and fulfilment of these KCs in medical training needs to be better studied from the undergraduate to expert level [41], which highlights that the assessment of these KCs, including those in the workplace, is essential and must be stimulated [44].

There are KCs, such as sharing in decision-making, that can be assessed and improved upon, but it is necessary to acknowledge that some level of excellence or competency could be unattainable [29]. Some of the actual and traditional evaluation methods have been insufficient to assess emergent outcomes for students’ leadership, healthcare improvement and other skills [45], and the assessment of more complex competencies requires well-designed programmes [46]. The psychometric properties of rating scales for communication are mainly intermediate, even in a more controlled environment, like objective structured clinical exams [44]. Therefore, it is important to improve assessment methods for all KCs regardless of when they are achieved.

Although it is difficult to ensure that all of these KCs are achieved during undergraduate study, it is important to do so. This would also serve to support efforts made to overcome the prejudice of them being considered as personal characteristics or minor competencies; instead, medical educators must strive to value, teach and assess them correctly.

### Was the time to achieve competencies influenced by the participants’ personal characteristics?

A medical educator’s viewpoint can be affected not only by academic evidence and standards but also by experiences and individual beliefs [47]. The participants in the current study tended to suggest a time for the attainment of competencies around the same time they believed they had ideally attained them. The completion of a residency in family medicine, the number of years spent working as a preceptor and the number of years spent working as a faculty member influenced the subjects’ opinions regarding the optimal time frame for achievement of a KC.

KCs for CCS can be understood as being among the most important and challenging competencies to be developed in practice [4]; these competencies underpin the doctor–patient relationship and are linked with a physician’s performance and outcomes [48]. In contrast to non-specialists, subjects who had completed a family medicine residency tended to report that CCS should be achieved later. The need for CCS skills is profoundly increased during a family medicine residency [49]. Therefore, the relevance and high stakes of CCS may explain why the participants suggested the achievement of these competencies at later periods.

The preceptors focused on providing healthcare to patients, promoting the use of a real world environment for medical students, working with technical and ethical developments in the workplace [50] and integrating healthcare skills and knowledge with education [51]. They also focused on placing students in interdisciplinary situations, working with members of various professions and introducing students and residents to this environment [51]. These professionals have experience in Leadership and IC and are probably among the best candidates to contribute to strategies for teaching these competencies. However, the preceptors’ needs to develop high standards of IC may have driven them to indicate that these KCs should be achieved later.

**Fig. 1:**
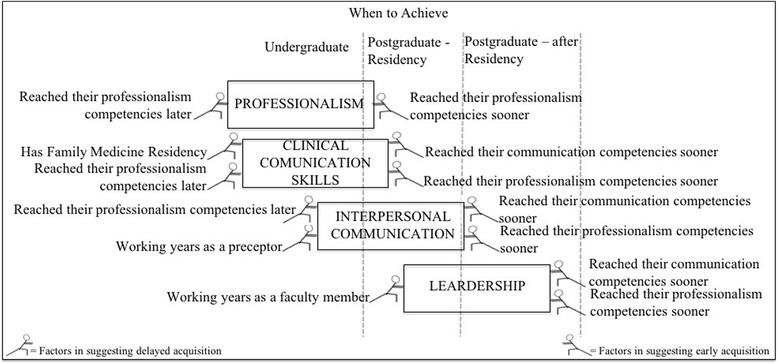
Factors influencing the subjects’ points of view of when students should achieve the competencies

The degree of each participant’s competence was not assessed, and the competencies and levels of expertise for each KC certainly differed among the subjects. This must also have come into play in the results that indicated that the medical educators’ attributes influenced their opinion on when they proposed that a competency should be achieved. The elements that influenced the participants’ points of views on when students should attain these competencies are shown in Fig. 1.

### Limitations of the study

There are two main limitations to the current study: one is the representativeness of the sample’s subjects and the other is the difficulty of guaranteeing that each competency was defined in the same way by all the participants.

A definition for ‘competency’ was not provided, and, because there is some variety in specific definitions for this term, the meaning could have varied among the participants. Another point is that even having proceeded carefully in the thematic organisation, to define the KCs, there were some KC’s that were open to various levels of personal interpretation. These KCs, therefore, can be difficult to measure and assess [44]. In addition, aside from personal interpretations, there was also the complexity of assessing and judging the level of achievement required for a KC in order to work as a physician, with the duties and responsibilities of the profession [52]. These biases in the definition of assessment and attainment could blur viewpoints about a competency and the best time frame for its achievement. The subject’s point of view can be based not only on competencies that they consider fundamental to undergraduate students but also on their opinion of the junior doctors or registrars they supervise in their practices who have not achieved these competencies.

Considering the continental dimensions and number of medical schools in Brazil (272) [53], the 74 participants in the current study were not a representative sample of all the medical teachers in the country. However, the statistical significance for almost all of the competencies and subdomains was good and represented the opinions of this group of family physicians’ medical educators. The mean age was 37.9, which is younger than in other samples of medical educators in Brazil [54]. However, the fact that residency programmes in family medicine, the inclusion of the discipline of family medicine in medical schools and the mastery of these doctors in medical education has become more important in Brazil in the last several years [55–57] explains the youth of the sample. Another possible bias is that family physicians frequently have specific training in communication competencies.

Therefore, these results must be analysed in light of these limitations, including, that doctors from other specialties could have different points of view.

## Conclusion

It was possible to suggest an order and time frame for the development of communication and professionalism KCs during medical training. The KCs for CCS and Professionalism were indicated as needing to be achieved sooner. Following those were the IC and Leadership KCs, the basis of which should be formed at the undergraduate level. This is because the mastery of IC and Leadership competencies demands a profound immersion in workplace and medical responsibilities.

The influence of the participants’ professional experiences on their viewpoints regarding the achievement of the KCs showed that the medical educators’ opinions could have been driven not only by academic knowledge of medical education but also by their own perceived personal development. On one hand, this influence can be considered biased, but on the other hand, it can be thought of as a more realistic viewpoint because it comes from people who are deeply immersed in the field.

The opinions of these family physicians’ medical educators can assist with the development of required outcomes for medical training, which could drive the organisation of medical curricula and support programmes of lifelong learning. The certification of these KCs and improvement in assessment methods, including their impact on healthcare, are the next steps for future research.

## Additional file

Additional file 1: Survey Questionnaire. (PDF 184 kb)

## Abbreviations

CCS: clinical communication skills
IC: interpersonal communication
KCs: key competencies
