# Supplementary material for: Clinical communication skills and professionalism education are required from the beginning of medical training - a point of view of family physicians
Source: BMC Med Educ. 2018 Mar 20;18:43. doi: 10.1186/s12909-018-1141-2 (PMC5859538; doi:10.1186/s12909-018-1141-2)
Supplement: Supplementary file 1 — Survey Questionnaire. (PDF 184 kb) [file 12909_2018_1141_MOESM1_ESM.pdf]

COMPETENCY SURVEY

Please indicate your graduate year and age.

Below, please check the stage of YOUR training when your competencies developed sufficiently for the practice of medicine.

	Undergraduate	Residency	After Residency	Others
Communication Skills	( )	( )	( )	( )
Professionalism	( )	( )	( )	( )

What activities have you already done or currently do? Teaching: activities with students that include the realisation of teaching plans, e.g. classes, seminars or evaluations, with or without formal contact with educational institutions. Preceptory: activities essentially linked to practice and internships in the field, without performing functions as a doctor, and accompanying and training undergraduate students, in this case, without responsibility for teaching pedagogical planning).

	Yes	No
Clinical Activity	( )	( )
Teaching	( )	( )
Preceptory	( )	( )
Other	( )	( )

How long in the activity? (Clinical)
How long in the activity? (Teaching)
How long in the activity? (Preceptor)
How long in the activity? (Other)

What academic degrees do you have? (Check more than one option if needed)

( ) Undergraduate; ( ) Residency in Family Medice; ( ) Specialty - Title; ( ) Master’s Degree; ( ) Doctoral Degree, ( ) Post-Doctoral Degree, ( ) Others

Do you have any other undergraduate qualifications other than medicine?

For the competencies listed below, when should students adequately achieve each competency?

Competency	Undergraduate	FM Residency	After Residency
Communicate effectively according to given roles	( )	( )	( )
Establish a therapeutic and professional relationship	( )	( )	( )
Build a suitable relationship	( )	( )	( )
Involve the bio-psycho-social context	( )	( )	( )
Understand the perspective of the patient and his or her family	( )	( )	( )
Adapt communication according to the patient and his or her family	( )	( )	( )
Engage patients and families to share in decision-making	( )	( )	( )
Support decision-making based on the needs and interests of the patient	( )	( )	( )
Structure and organize communication/clinical interviews	( )	( )	( )
Communicate bad news appropriately	( )	( )	( )
Inform patients and family adequately	( )	( )	( )
None of these Key Competencies	( )	( )	( )
Perform consulting, helping colleagues, other professionals, and the healthcare system	( )	( )	( )
Communicate effectively to promote understanding and resolve conflicts, aiming to ensure the success of teamwork	( )	( )	( )
Perform teamwork, aiming to ensure patient safety	( )	( )	( )
Communicate about ethical issues with other health professionals	( )	( )	( )
Demonstrate basic leadership skills	( )	( )	( )
Engage in the management of human and health care resources	( )	( )	( )
None of these Key Competencies	( )	( )	( )
Act with interest and dedication	( )	( )	( )
Prioritize patient's/family's/community's interests above one's own	( )	( )	( )
Recognize one's limits and know when to request support	( )	( )	( )
Be responsible and careful in one's actions	( )	( )	( )
Attempt to promote patient and/or family safety	( )	( )	( )
Act according to the highest standards of excellence and know where to seek knowledge	( )	( )	( )
Be empathic and respectful, valuing the feelings and wishes of colleagues, patients, teachers, and other professionals	( )	( )	( )
Consider the beliefs, needs, and views of patients/families	( )	( )	( )
Reflect and have good critical skills	( )	( )	( )
Deal with uncertainty appropriately, adapting to different situations and contexts	( )	( )	( )
Recognize and nurture their own physical and mental health	( )	( )	( )

ABOUT THE NEXT QUESTIONS

The next questions were constructed from medical education fragments by consensus. The following were used: CanMed, ACGME, Tomorrow's Doctors, the Australian Medical Council, Basel (Guideline of the countries of the lingua franca German-Communication Competence), the Calgary Cambridge (Clinical Communication Directive), the Kalamazoo Consensus (Communication Competency Directive) and HPCC (European Consensus on Communication Skills).

These consensuses defined the competencies. The intention was from the point of view of family physicians and the community to organize these skills in general or common domains of competencies (key competencies). The focus was the construction of these general domains for competence of clinical communication and professionalism. This made it easier and more practical to include specific skills, since these larger areas were already organized.

Two researchers independently analysed the above documents and agreed on 18 general domains for communication competence (key competencies).

In the first step of the questionnaire, the fragments of the documents are arranged, and between the options will be the domains that the researchers defined by consensus.

You will point out in which of these domains that the fragments fit. If a fragment does not fit at all, check the 'other' alternative.

Be aware that the fragments must be contained in the domains (totally or in part), the domains being more representative, or more global, than the fragments or its main idea.

( ) I have read and understood

( ) I have read and not understand

Do you have any question or doubts?

For the competency, "communicates effectively with patients, families and the public", which is the key competency that best represents its meaning?

Communicate effectively according to given roles	( )
Establish a therapeutic and professional relationship	( )
Build a suitable relationship	( )
Involve the bio-psycho-social context	( )
Understand the perspective of the patient and his or her family	( )
Adapt communication according to the patient and his or her family	( )
Engage patients and families to share in decision-making	( )
Support decision-making based on the needs and interests of the patient	( )
Structure and organize communication/clinical interviews	( )
Communicate bad news appropriately	( )
Inform patients and family adequately	( )
None of these Key Competencies	( )

For the competency: "Communicate effectively in wider roles including health advocacy, teaching, assessing and appraising. Communicate effectively about ethical issues with patients and family ", which is the key competency that best represents its meaning?

Communicate effectively according to given roles	( )
Establish a therapeutic and professional relationship	( )
Build a suitable relationship	( )
Involve the bio-psycho-social context	( )
Understand the perspective of the patient and his or her family	( )
Adapt communication according to the patient and his or her family	( )
Engage patients and families to share in decision-making	( )
Support decision-making based on the needs and interests of the patient	( )
Structure and organize communication/clinical interviews	( )
Communicate bad news appropriately	( )
Inform patients and family adequately	( )
None of these Key Competencies	( )

For the competency: "Communicate effectively in various roles, for example, as patient advocate, teacher, manager or improvement leader ", which is the key competency that best represents its meaning?

Communicate effectively according to given roles	( )
Establish a therapeutic and professional relationship	( )
Build a suitable relationship	( )
Involve the bio-psycho-social context	( )
Understand the perspective of the patient and his or her family	( )
Adapt communication according to the patient and his or her family	( )
Engage patients and families to share in decision-making	( )
Support decision-making based on the needs and interests of the patient	( )
Structure and organize communication/clinical interviews	( )
Communicate bad news appropriately	( )
Inform patients and family adequately	( )
None of these Key Competencies	( )

For the competency:"BUILDING RELATIONSHIP (Using appropriate non-verbal behaviour, Developing rapport and Involving the patient)", which is the key competency that best represents its meaning?

Communicate effectively according to given roles	( )
Establish a therapeutic and professional relationship	( )
Build a suitable relationship	( )
Involve the bio-psycho-social context	( )
Understand the perspective of the patient and his or her family	( )
Adapt communication according to the patient and his or her family	( )
Engage patients and families to share in decision-making	( )
Support decision-making based on the needs and interests of the patient	( )
Structure and organize communication/clinical interviews	( )
Communicate bad news appropriately	( )
Inform patients and family adequately	( )
None of these Key Competencies	( )

For the competency:"uses effective and efficient communication and management strategies", which is the key competency that best represents its meaning?

Communicate effectively according to given roles	( )
Establish a therapeutic and professional relationship	( )
Build a suitable relationship	( )
Involve the bio-psycho-social context	( )
Understand the perspective of the patient and his or her family	( )
Adapt communication according to the patient and his or her family	( )

Engage patients and families to share in decision-making	( )
Support decision-making based on the needs and interests of the patient	( )
Structure and organize communication/clinical interviews	( )
Communicate bad news appropriately	( )
Inform patients and family adequately	( )
None of these Key Competencies	( )

For the competency:"Shaping of relationship: involves the patient in the interaction using a patient-centred approach.", which is the key competency that best represents its meaning?

Communicate effectively according to given roles	( )
Establish a therapeutic and professional relationship	( )
Build a suitable relationship	( )
Involve the bio-psycho-social context	( )
Understand the perspective of the patient and his or her family	( )
Adapt communication according to the patient and his or her family	( )
Engage patients and families to share in decision-making	( )
Support decision-making based on the needs and interests of the patient	( )
Structure and organize communication/clinical interviews	( )
Communicate bad news appropriately	( )
Inform patients and family adequately	( )
None of these Key Competencies	( )

For the competency:"Involve patients in decision-making and planning their treatment, including communicating risk and benefits of management options.", which is the key competency that best represents its meaning?

Communicate effectively according to given roles	( )
Establish a therapeutic and professional relationship	( )
Build a suitable relationship	( )
Involve the bio-psycho-social context	( )
Understand the perspective of the patient and his or her family	( )
Adapt communication according to the patient and his or her family	( )
Engage patients and families to share in decision-making	( )
Support decision-making based on the needs and interests of the patient	( )
Structure and organize communication/clinical interviews	( )
Communicate bad news appropriately	( )
Inform patients and family adequately	( )
None of these Key Competencies	( )

For the competency:"Engage patients and their families...", which is the key competency that best represents its meaning?

Communicate effectively according to given roles	( )
Establish a therapeutic and professional relationship	( )
Build a suitable relationship	( )
Involve the bio-psycho-social context	( )
Understand the perspective of the patient and his or her family	( )
Adapt communication according to the patient and his or her family	( )
Engage patients and families to share in decision-making	( )
Support decision-making based on the needs and interests of the patient	( )
Structure and organize communication/clinical interviews	( )
Communicate bad news appropriately	( )
Inform patients and family adequately	( )
None of these Key Competencies	( )

For the competency:"involves the patient in the interaction to establish a therapeutic relationship using a patient-centered approach.", which is the key competency that best represents its meaning?

Communicate effectively according to given roles	( )
Establish a therapeutic and professional relationship	( )
Build a suitable relationship	( )
Involve the bio-psycho-social context	( )
Understand the perspective of the patient and his or her family	( )
Adapt communication according to the patient and his or her family	( )
Engage patients and families to share in decision-making	( )
Support decision-making based on the needs and interests of the patient	( )
Structure and organize communication/clinical interviews	( )
Communicate bad news appropriately	( )
Inform patients and family adequately	( )
None of these Key Competencies	( )

For the competency:"GATHERING INFORMATION (Exploration of patient's problems and Additional skills for understanding the patient's perspective).", which is the key competency that best represents its meaning?

Communicate effectively according to given roles	( )
Establish a therapeutic and professional relationship	( )
Build a suitable relationship	( )
Involve the bio-psycho-social context	( )
Understand the perspective of the patient and his or her family	( )

Adapt communication according to the patient and his or her family	( )
Engage patients and families to share in decision-making	( )
Support decision-making based on the needs and interests of the patient	( )
Structure and organize communication/clinical interviews	( )
Communicate bad news appropriately	( )
Inform patients and family adequately	( )
None of these Key Competencies	( )

For the competency:"Gather information", which is the key competency that best represents its meaning?

Communicate effectively according to given roles	( )
Establish a therapeutic and professional relationship	( )
Build a suitable relationship	( )
Involve the bio-psycho-social context	( )
Understand the perspective of the patient and his or her family	( )
Adapt communication according to the patient and his or her family	( )
Engage patients and families to share in decision-making	( )
Support decision-making based on the needs and interests of the patient	( )
Structure and organize communication/clinical interviews	( )
Communicate bad news appropriately	( )
Inform patients and family adequately	( )
None of these Key Competencies	( )

For the competency:"...share information", which is the key competency that best represents its meaning?

Communicate effectively according to given roles	( )
Establish a therapeutic and professional relationship	( )
Build a suitable relationship	( )
Involve the bio-psycho-social context	( )
Understand the perspective of the patient and his or her family	( )
Adapt communication according to the patient and his or her family	( )
Engage patients and families to share in decision-making	( )
Support decision-making based on the needs and interests of the patient	( )
Structure and organize communication/clinical interviews	( )
Communicate bad news appropriately	( )
Inform patients and family adequately	( )
None of these Key Competencies	( )

For the competency:"Communication in the doctor–patient relationship: orients her communication behaviour along the actual concerns and the personality of the patient", which is the key competency that best represents its meaning?

Communicate effectively according to given roles	( )
Establish a therapeutic and professional relationship	( )
Build a suitable relationship	( )
Involve the bio-psycho-social context	( )
Understand the perspective of the patient and his or her family	( )
Adapt communication according to the patient and his or her family	( )
Engage patients and families to share in decision-making	( )
Support decision-making based on the needs and interests of the patient	( )
Structure and organize communication/clinical interviews	( )
Communicate bad news appropriately	( )
Inform patients and family adequately	( )
None of these Key Competencies	( )

For the competency:"...recognizes the patient as a partner in shaping a relationship", which is the key competency that best represents its meaning?

Communicate effectively according to given roles	( )
Establish a therapeutic and professional relationship	( )
Build a suitable relationship	( )
Involve the bio-psycho-social context	( )
Understand the perspective of the patient and his or her family	( )
Adapt communication according to the patient and his or her family	( )
Engage patients and families to share in decision-making	( )
Support decision-making based on the needs and interests of the patient	( )
Structure and organize communication/clinical interviews	( )
Communicate bad news appropriately	( )
Inform patients and family adequately	( )
None of these Key Competencies	( )

For the competency:"Relates to the patient respectfully including ensuring confidentiality, privacy and autonomy...", which is the key competency that best represents its meaning?

Communicate effectively according to given roles	( )
Establish a therapeutic and professional relationship	( )
Build a suitable relationship	( )
Involve the bio-psycho-social context	( )
Understand the perspective of the patient and his or her family	( )
Adapt communication according to the patient and his or her family	( )

Engage patients and families to share in decision-making	( )
Support decision-making based on the needs and interests of the patient	( )
Structure and organize communication/clinical interviews	( )
Communicate bad news appropriately	( )
Inform patients and family adequately	( )
None of these Key Competencies	( )

For the competency:"Elicits and explores the content of the patient's bio-psycho-social history.", which is the key competency that best represents its meaning?

Communicate effectively according to given roles	( )
Establish a therapeutic and professional relationship	( )
Build a suitable relationship	( )
Involve the bio-psycho-social context	( )
Understand the perspective of the patient and his or her family	( )
Adapt communication according to the patient and his or her family	( )
Engage patients and families to share in decision-making	( )
Support decision-making based on the needs and interests of the patient	( )
Structure and organize communication/clinical interviews	( )
Communicate bad news appropriately	( )
Inform patients and family adequately	( )
None of these Key Competencies	( )

For the competency:"Encouragethe patient to express own ideas, concers, expectations and feelings and accepts legitimacy of patients views and feeling", which is the key competency that best represents its meaning?

Communicate effectively according to given roles	( )
Establish a therapeutic and professional relationship	( )
Build a suitable relationship	( )
Involve the bio-psycho-social context	( )
Understand the perspective of the patient and his or her family	( )
Adapt communication according to the patient and his or her family	( )
Engage patients and families to share in decision-making	( )
Support decision-making based on the needs and interests of the patient	( )
Structure and organize communication/clinical interviews	( )
Communicate bad news appropriately	( )
Inform patients and family adequately	( )
None of these Key Competencies	( )

For the competency:"...developing plans that reflect the patient's health care needs and goals", which is the key competency that best represents its meaning?

Communicate effectively according to given roles	( )

Establish a therapeutic and professional relationship	( )
Build a suitable relationship	( )
Involve the bio-psycho-social context	( )
Understand the perspective of the patient and his or her family	( )
Adapt communication according to the patient and his or her family	( )
Engage patients and families to share in decision-making	( )
Support decision-making based on the needs and interests of the patient	( )
Structure and organize communication/clinical interviews	( )
Communicate bad news appropriately	( )
Inform patients and family adequately	( )
None of these Key Competencies	( )

For the competency:"Social behaviour and communication: adapts her social behaviour and communication to different social contexts and communication partners.", which is the key competency that best represents its meaning?

Communicate effectively according to given roles	( )
Establish a therapeutic and professional relationship	( )
Build a suitable relationship	( )
Involve the bio-psycho-social context	( )
Understand the perspective of the patient and his or her family	( )
Adapt communication according to the patient and his or her family	( )
Engage patients and families to share in decision-making	( )
Support decision-making based on the needs and interests of the patient	( )
Structure and organize communication/clinical interviews	( )
Communicate bad news appropriately	( )
Inform patients and family adequately	( )
None of these Key Competencies	( )

For the competency:"adapts own communication to the level of understanding and language of the patient, uses techniques aproppriates for this.", which is the key competency that best represents its meaning?

Communicate effectively according to given roles	( )
Establish a therapeutic and professional relationship	( )
Build a suitable relationship	( )
Involve the bio-psycho-social context	( )
Understand the perspective of the patient and his or her family	( )
Adapt communication according to the patient and his or her family	( )
Engage patients and families to share in decision-making	( )
Support decision-making based on the needs and interests of the patient	( )
Structure and organize communication/clinical interviews	( )
Communicate bad news appropriately	( )

Inform patients and family adequately	( )
None of these Key Competencies	( )

For the competency:"EXPLANATION AND PLANNING (Providing the correct amount and type of information, Aiding accurate recall and understanding...)", which is the key competency that best represents its meaning?

Communicate effectively according to given roles	( )
Establish a therapeutic and professional relationship	( )
Build a suitable relationship	( )
Involve the bio-psycho-social context	( )
Understand the perspective of the patient and his or her family	( )
Adapt communication according to the patient and his or her family	( )
Engage patients and families to share in decision-making	( )
Support decision-making based on the needs and interests of the patient	( )
Structure and organize communication/clinical interviews	( )
Communicate bad news appropriately	( )
Inform patients and family adequately	( )
None of these Key Competencies	( )

For the competency:"EXPLANATION AND PLANNING (Achieving a shared understanding: incorporating the patient’s perspective and Planning: shared decision making)", which is the key competency that best represents its meaning?

Communicate effectively according to given roles	( )
Establish a therapeutic and professional relationship	( )
Build a suitable relationship	( )
Involve the bio-psycho-social context	( )
Understand the perspective of the patient and his or her family	( )
Adapt communication according to the patient and his or her family	( )
Engage patients and families to share in decision-making	( )
Support decision-making based on the needs and interests of the patient	( )
Structure and organize communication/clinical interviews	( )
Communicate bad news appropriately	( )
Inform patients and family adequately	( )
None of these Key Competencies	( )

For the competency: "Understand the Patient’s Perspective", which is the key competency that best represents its meaning?

Communicate effectively according to given roles	( )
Establish a therapeutic and professional relationship	( )
Build a suitable relationship	( )
Involve the bio-psycho-social context	( )
Understand the perspective of the patient and his or her family	( )

Adapt communication according to the patient and his or her family	( )
Engage patients and families to share in decision-making	( )
Support decision-making based on the needs and interests of the patient	( )
Structure and organize communication/clinical interviews	( )
Communicate bad news appropriately	( )
Inform patients and family adequately	( )
None of these Key Competencies	( )

For the competency: "Share health care information and plans with patients and their families", which is the key competency that best represents its meaning?

Communicate effectively according to given roles	( )
Establish a therapeutic and professional relationship	( )
Build a suitable relationship	( )
Involve the bio-psycho-social context	( )
Understand the perspective of the patient and his or her family	( )
Adapt communication according to the patient and his or her family	( )
Engage patients and families to share in decision-making	( )
Support decision-making based on the needs and interests of the patient	( )
Structure and organize communication/clinical interviews	( )
Communicate bad news appropriately	( )
Inform patients and family adequately	( )
None of these Key Competencies	( )

For the competency: "Demonstrate by listening, sharing and responding, the ability to communicate clearly, sensitively and effectively with patients and their family/carers", which is the key competency that best represents its meaning?

Communicate effectively according to given roles	( )
Establish a therapeutic and professional relationship	( )
Build a suitable relationship	( )
Involve the bio-psycho-social context	( )
Understand the perspective of the patient and his or her family	( )
Adapt communication according to the patient and his or her family	( )
Engage patients and families to share in decision-making	( )
Support decision-making based on the needs and interests of the patient	( )
Structure and organize communication/clinical interviews	( )
Communicate bad news appropriately	( )
Inform patients and family adequately	( )
None of these Key Competencies	( )

For the competency: "Communicate clearly, sensitively and effectively with patients, their relatives or other carers, and colleagues from the medical and other professions, by

listening, sharing and responding", which is the key competency that best represents its meaning?

Communicate effectively according to given roles	( )
Establish a therapeutic and professional relationship	( )
Build a suitable relationship	( )
Involve the bio-psycho-social context	( )
Understand the perspective of the patient and his or her family	( )
Adapt communication according to the patient and his or her family	( )
Engage patients and families to share in decision-making	( )
Support decision-making based on the needs and interests of the patient	( )
Structure and organize communication/clinical interviews	( )
Communicate bad news appropriately	( )
Inform patients and family adequately	( )
None of these Key Competencies	( )

For the competency: "Information: effectively collects the relevant information for the reasoning and decision-making process", which is the key competency that best represents its meaning?

Communicate effectively according to given roles	( )
Establish a therapeutic and professional relationship	( )
Build a suitable relationship	( )
Involve the bio-psycho-social context	( )
Understand the perspective of the patient and his or her family	( )
Adapt communication according to the patient and his or her family	( )
Engage patients and families to share in decision-making	( )
Support decision-making based on the needs and interests of the patient	( )
Structure and organize communication/clinical interviews	( )
Communicate bad news appropriately	( )
Inform patients and family adequately	( )
None of these Key Competencies	( )

For the competency: "Information: effectively communicates the relevant information for the reasoning and decision-making process", which is the key competency that best represents its meaning?

Communicate effectively according to given roles	( )
Establish a therapeutic and professional relationship	( )
Build a suitable relationship	( )
Involve the bio-psycho-social context	( )
Understand the perspective of the patient and his or her family	( )
Adapt communication according to the patient and his or her family	( )
Engage patients and families to share in decision-making	( )

Support decision-making based on the needs and interests of the patient	( )
Structure and organize communication/clinical interviews	( )
Communicate bad news appropriately	( )
Inform patients and family adequately	( )
None of these Key Competencies	( )

For the competency: "effectively collects relevant information for reasoning and decision making.", which is the key competency that best represents its meaning?

Communicate effectively according to given roles	( )
Establish a therapeutic and professional relationship	( )
Build a suitable relationship	( )
Involve the bio-psycho-social context	( )
Understand the perspective of the patient and his or her family	( )
Adapt communication according to the patient and his or her family	( )
Engage patients and families to share in decision-making	( )
Support decision-making based on the needs and interests of the patient	( )
Structure and organize communication/clinical interviews	( )
Communicate bad news appropriately	( )
Inform patients and family adequately	( )
None of these Key Competencies	( )

For the competency: "effectively communicates relevant information for reasoning and decision making. Gives information to the patient in a timely, comprehensive and meaningful manner", which is the key competency that best represents its meaning?

Communicate effectively according to given roles	( )
Establish a therapeutic and professional relationship	( )
Build a suitable relationship	( )
Involve the bio-psycho-social context	( )
Understand the perspective of the patient and his or her family	( )
Adapt communication according to the patient and his or her family	( )
Engage patients and families to share in decision-making	( )
Support decision-making based on the needs and interests of the patient	( )
Structure and organize communication/clinical interviews	( )
Communicate bad news appropriately	( )
Inform patients and family adequately	( )
None of these Key Competencies	( )

For the competency: "Establish professional therapeutic relationships with patients and their families", which is the key competency that best represents its meaning?

Communicate effectively according to given roles	( )
Establish a therapeutic and professional relationship	( )
Build a suitable relationship	( )

Involve the bio-psycho-social context	( )
Understand the perspective of the patient and his or her family	( )
Adapt communication according to the patient and his or her family	( )
Engage patients and families to share in decision-making	( )
Support decision-making based on the needs and interests of the patient	( )
Structure and organize communication/clinical interviews	( )
Communicate bad news appropriately	( )
Inform patients and family adequately	( )
None of these Key Competencies	( )

For the competency: "create and sustain a therapeutic, ethical relationships with patients", which is the key competency that best represents its meaning?

Communicate effectively according to given roles	( )
Establish a therapeutic and professional relationship	( )
Build a suitable relationship	( )
Involve the bio-psycho-social context	( )
Understand the perspective of the patient and his or her family	( )
Adapt communication according to the patient and his or her family	( )
Engage patients and families to share in decision-making	( )
Support decision-making based on the needs and interests of the patient	( )
Structure and organize communication/clinical interviews	( )
Communicate bad news appropriately	( )
Inform patients and family adequately	( )
None of these Key Competencies	( )

For the competency: "Demonstrate by listening, sharing and responding, the ability to communicate clearly, sensitively and effectively with patients and their family/carers", which is the key competency that best represents its meaning?

Communicate effectively according to given roles	( )
Establish a therapeutic and professional relationship	( )
Build a suitable relationship	( )
Involve the bio-psycho-social context	( )
Understand the perspective of the patient and his or her family	( )
Adapt communication according to the patient and his or her family	( )
Engage patients and families to share in decision-making	( )
Support decision-making based on the needs and interests of the patient	( )
Structure and organize communication/clinical interviews	( )
Communicate bad news appropriately	( )
Inform patients and family adequately	( )
None of these Key Competencies	( )

For the competency: "INITIATING THE SESSION (Establishing initial rapport and Identifying the reason(s) for the consultation)", which is the key competency that best represents its meaning?

Communicate effectively according to given roles	( )
Establish a therapeutic and professional relationship	( )
Build a suitable relationship	( )
Involve the bio-psycho-social context	( )
Understand the perspective of the patient and his or her family	( )
Adapt communication according to the patient and his or her family	( )
Engage patients and families to share in decision-making	( )
Support decision-making based on the needs and interests of the patient	( )
Structure and organize communication/clinical interviews	( )
Communicate bad news appropriately	( )
Inform patients and family adequately	( )
None of these Key Competencies	( )

For the competency: "Open the Discussion", which is the key competency that best represents its meaning?

Communicate effectively according to given roles	( )
Establish a therapeutic and professional relationship	( )
Build a suitable relationship	( )
Involve the bio-psycho-social context	( )
Understand the perspective of the patient and his or her family	( )
Adapt communication according to the patient and his or her family	( )
Engage patients and families to share in decision-making	( )
Support decision-making based on the needs and interests of the patient	( )
Structure and organize communication/clinical interviews	( )
Communicate bad news appropriately	( )
Inform patients and family adequately	( )
None of these Key Competencies	( )

For the competency: "Elicit and synthesize accurate and relevant information, incorporating the perspectives of patients and their families", which is the key competency that best represents its meaning?

Communicate effectively according to given roles	( )
Establish a therapeutic and professional relationship	( )
Build a suitable relationship	( )
Involve the bio-psycho-social context	( )
Understand the perspective of the patient and his or her family	( )
Adapt communication according to the patient and his or her family	( )

Engage patients and families to share in decision-making	( )
Support decision-making based on the needs and interests of the patient	( )
Structure and organize communication/clinical interviews	( )
Communicate bad news appropriately	( )
Inform patients and family adequately	( )
None of these Key Competencies	( )

For the competency: "Shapes a conversation from beginning to end with regard to structure (e.g. introduction, initiating the conversation, gathering and giving information, planning, closing interview, setting up next meeting; time management)", which is the key competency that best represents its meaning?

Communicate effectively according to given roles	( )
Establish a therapeutic and professional relationship	( )
Build a suitable relationship	( )
Involve the bio-psycho-social context	( )
Understand the perspective of the patient and his or her family	( )
Adapt communication according to the patient and his or her family	( )
Engage patients and families to share in decision-making	( )
Support decision-making based on the needs and interests of the patient	( )
Structure and organize communication/clinical interviews	( )
Communicate bad news appropriately	( )
Inform patients and family adequately	( )
None of these Key Competencies	( )

For the competency: "PROVIDING STRUCTURE (Making organisation overt And Attending to flow)", which is the key competency that best represents its meaning?

Communicate effectively according to given roles	( )
Establish a therapeutic and professional relationship	( )
Build a suitable relationship	( )
Involve the bio-psycho-social context	( )
Understand the perspective of the patient and his or her family	( )
Adapt communication according to the patient and his or her family	( )
Engage patients and families to share in decision-making	( )
Support decision-making based on the needs and interests of the patient	( )
Structure and organize communication/clinical interviews	( )
Communicate bad news appropriately	( )
Inform patients and family adequately	( )
None of these Key Competencies	( )

For the competency: "Communicate appropriately with difficult or violent patients; people with mental illness and vulnerable patients//Communicate appropriately in difficult circumstances, such as when breaking bad news, and when discussing sensitive

issues, such as alcohol consumption, smoking or obesity", which is the key competency that best represents its meaning?

Communicate effectively according to given roles	( )
Establish a therapeutic and professional relationship	( )
Build a suitable relationship	( )
Involve the bio-psycho-social context	( )
Understand the perspective of the patient and his or her family	( )
Adapt communication according to the patient and his or her family	( )
Engage patients and families to share in decision-making	( )
Support decision-making based on the needs and interests of the patient	( )
Structure and organize communication/clinical interviews	( )
Communicate bad news appropriately	( )
Inform patients and family adequately	( )
None of these Key Competencies	( )

For the competency: "Recognizes difficult situations and communication challenges and deals with them sensitively and constructively", which is the key competency that best represents its meaning?

Communicate effectively according to given roles	( )
Establish a therapeutic and professional relationship	( )
Build a suitable relationship	( )
Involve the bio-psycho-social context	( )
Understand the perspective of the patient and his or her family	( )
Adapt communication according to the patient and his or her family	( )
Engage patients and families to share in decision-making	( )
Support decision-making based on the needs and interests of the patient	( )
Structure and organize communication/clinical interviews	( )
Communicate bad news appropriately	( )
Inform patients and family adequately	( )
None of these Key Competencies	( )

For the competency: "communicate effectively with physicians, other health professionals and health-related agencies", which is the key competency that best represents its meaning?

Work effectively in multidisciplinary team, adapting to the particularities of each team and given roles	( )
Perform consulting, helping colleagues, other professionals, and the healthcare system	( )
Communicate effectively to promote understanding and resolve conflicts, aiming to ensure the success of teamwork	( )
Perform teamwork, aiming to ensure patient safety	( )
Communicate about ethical issues with other health professionals	( )
Demonstrate basic leadership skills	( )
Engage in the management of human and health care resources	( )
None of these Key Competencies	( )

For the competency: "Team building and working in a team: adapts her behaviour to different phases of team building and efficiently shapes her working style to contribute to a successful team", which is the key competency that best represents its meaning?

Work effectively in multidisciplinary team, adapting to the particularities of each team and given roles	( )
Perform consulting, helping colleagues, other professionals, and the healthcare system	( )
Communicate effectively to promote understanding and resolve conflicts, aiming to ensure the success of teamwork	( )
Perform teamwork, aiming to ensure patient safety	( )
Communicate about ethical issues with other health professionals	( )
Demonstrate basic leadership skills	( )
Engage in the management of human and health care resources	( )
None of these Key Competencies	( )

For the competency: "shows ability to communicate effectively in multi-professional teams", which is the key competency that best represents its meaning?

Work effectively in multidisciplinary team, adapting to the particularities of each team and given roles	( )
Perform consulting, helping colleagues, other professionals, and the healthcare system	( )
Communicate effectively to promote understanding and resolve conflicts, aiming to ensure the success of teamwork	( )
Perform teamwork, aiming to ensure patient safety	( )
Communicate about ethical issues with other health professionals	( )
Demonstrate basic leadership skills	( )
Engage in the management of human and health care resources	( )
None of these Key Competencies	( )

For the competency: "act in a consultative role to other physicians, health-related

agencies and policy-makers", which is the key competency that best represents its meaning?

Work effectively in multidisciplinary team, adapting to the particularities of each team and given roles	( )
Perform consulting, helping colleagues, other professionals, and the healthcare system	( )
Communicate effectively to promote understanding and resolve conflicts, aiming to ensure the success of teamwork	( )
Perform teamwork, aiming to ensure patient safety	( )
Communicate about ethical issues with other health professionals	( )
Demonstrate basic leadership skills	( )
Engage in the management of human and health care resources	( )
None of these Key Competencies	( )

For the competency: "Contribute to the improvement of health care delivery in teams, organizations, and systems", which is the key competency that best represents its meaning?

Work effectively in multidisciplinary team, adapting to the particularities of each team and given roles	( )
Perform consulting, helping colleagues, other professionals, and the healthcare system	( )
Communicate effectively to promote understanding and resolve conflicts, aiming to ensure the success of teamwork	( )
Perform teamwork, aiming to ensure patient safety	( )
Communicate about ethical issues with other health professionals	( )
Demonstrate basic leadership skills	( )
Engage in the management of human and health care resources	( )
None of these Key Competencies	( )

For the competency: "Demonstrate by listening, sharing and responding, the ability to communicate clearly, sensitively and effectively with doctors and other health professionals.", which is the key competency that best represents its meaning?

Work effectively in multidisciplinary team, adapting to the particularities of each team and given roles	( )
Perform consulting, helping colleagues, other professionals, and the healthcare system	( )
Communicate effectively to promote understanding and resolve conflicts, aiming to ensure the success of teamwork	( )
Perform teamwork, aiming to ensure patient safety	( )
Communicate about ethical issues with other health professionals	( )
Demonstrate basic leadership skills	( )
Engage in the management of human and health care resources	( )
None of these Key Competencies	( )

For the competency: "Hand over the care of a patient to another health care professional

to facilitate continuity of safe patient care", which is the key competency that best represents its meaning?

Work effectively in multidisciplinary team, adapting to the particularities of each team and given roles	( )
Perform consulting, helping colleagues, other professionals, and the healthcare system	( )
Communicate effectively to promote understanding and resolve conflicts, aiming to ensure the success of teamwork	( )
Perform teamwork, aiming to ensure patient safety	( )
Communicate about ethical issues with other health professionals	( )
Demonstrate basic leadership skills	( )
Engage in the management of human and health care resources	( )
None of these Key Competencies	( )

For the competency: "Communicate effectively about ethical issues with health care professionals.", which is the key competency that best represents its meaning?

Work effectively in multidisciplinary team, adapting to the particularities of each team and given roles	( )
Perform consulting, helping colleagues, other professionals, and the healthcare system	( )
Communicate effectively to promote understanding and resolve conflicts, aiming to ensure the success of teamwork	( )
Perform teamwork, aiming to ensure patient safety	( )
Communicate about ethical issues with other health professionals	( )
Demonstrate basic leadership skills	( )
Engage in the management of human and health care resources	( )
None of these Key Competencies	( )

For the competency: "Work with physicians and other colleagues in the health care professions to promote understanding, manage differences, and resolve conflicts"; what is the key competency that best represents its meaning

Work effectively in multidisciplinary team, adapting to the particularities of each team and given roles	( )
Perform consulting, helping colleagues, other professionals, and the healthcare system	( )
Communicate effectively to promote understanding and resolve conflicts, aiming to ensure the success of teamwork	( )
Perform teamwork, aiming to ensure patient safety	( )
Communicate about ethical issues with other health professionals	( )
Demonstrate basic leadership skills	( )
Engage in the management of human and health care resources	( )
None of these Key Competencies	( )

For the competency: "Work effectively with physicians and other colleagues in the health care professions", which is the key competency that best represents its meaning?

Work effectively in multidisciplinary team, adapting to the particularities of each team and given roles	( )
Perform consulting, helping colleagues, other professionals, and the healthcare system	( )
Communicate effectively to promote understanding and resolve conflicts, aiming to ensure the success of teamwork	( )
Perform teamwork, aiming to ensure patient safety	( )
Communicate about ethical issues with other health professionals	( )
Demonstrate basic leadership skills	( )
Engage in the management of human and health care resources	( )
None of these Key Competencies	( )

For the competency: "Demonstrate leadership in professional practice .", which is the key competency that best represents its meaning?

Work effectively in multidisciplinary team, adapting to the particularities of each team and given roles	( )
Perform consulting, helping colleagues, other professionals, and the healthcare system	( )
Communicate effectively to promote understanding and resolve conflicts, aiming to ensure the success of teamwork	( )
Perform teamwork, aiming to ensure patient safety	( )
Communicate about ethical issues with other health professionals	( )
Demonstrate basic leadership skills	( )
Engage in the management of human and health care resources	( )
None of these Key Competencies	( )

For the competency: "work with other care providers as a team leader or member .", which is the key competency that best represents its meaning?

Work effectively in multidisciplinary team, adapting to the particularities of each team and given roles	( )
Perform consulting, helping colleagues, other professionals, and the healthcare system	( )
Communicate effectively to promote understanding and resolve conflicts, aiming to ensure the success of teamwork	( )
Perform teamwork, aiming to ensure patient safety	( )
Communicate about ethical issues with other health professionals	( )
Demonstrate basic leadership skills	( )
Engage in the management of human and health care resources	( )
None of these Key Competencies	( )

For the competency: "Describe the principles and practice of leadership in health care.", which is the key competency that best represents its meaning?

Work effectively in multidisciplinary team, adapting to the particularities of each team and given roles	( )
Perform consulting, helping colleagues, other professionals, and the healthcare system	( )
Communicate effectively to promote understanding and resolve conflicts, aiming to ensure the success of teamwork	( )
Perform teamwork, aiming to ensure patient safety	( )
Communicate about ethical issues with other health professionals	( )
Demonstrate basic leadership skills	( )
Engage in the management of human and health care resources	( )
None of these Key Competencies	( )

For the competency: "Leadership: shows basic competencies in leadership skills and supports the development and maintenance of the teamwork with her behaviour ", which is the key competency that best represents its meaning?

Work effectively in multidisciplinary team, adapting to the particularities of each team and given roles	( )
Perform consulting, helping colleagues, other professionals, and the healthcare system	( )
Communicate effectively to promote understanding and resolve conflicts, aiming to ensure the success of teamwork	( )
Perform teamwork, aiming to ensure patient safety	( )
Communicate about ethical issues with other health professionals	( )
Demonstrate basic leadership skills	( )
Engage in the management of human and health care resources	( )
None of these Key Competencies	( )

For the competency: "shows basic competencies in leadership skills ", which is the key competency that best represents its meaning?

Work effectively in multidisciplinary team, adapting to the particularities of each team and given roles	( )
Perform consulting, helping colleagues, other professionals, and the healthcare system	( )
Communicate effectively to promote understanding and resolve conflicts, aiming to ensure the success of teamwork	( )
Perform teamwork, aiming to ensure patient safety	( )
Communicate about ethical issues with other health professionals	( )
Demonstrate basic leadership skills	( )
Engage in the management of human and health care resources	( )
None of these Key Competencies	( )

For the competency: "Engage in the stewardship of health care resources ", which is the key competency that best represents its meaning?

Work effectively in multidisciplinary team, adapting to the particularities of each team and given roles	( )
Perform consulting, helping colleagues, other professionals, and the healthcare system	( )
Communicate effectively to promote understanding and resolve conflicts, aiming to ensure the success of teamwork	( )
Perform teamwork, aiming to ensure patient safety	( )
Communicate about ethical issues with other health professionals	( )
Demonstrate basic leadership skills	( )
Engage in the management of human and health care resources	( )
None of these Key Competencies	( )

For the competency: "Manage career planning, finances, and health human resources in a practice", which is the key competency that best represents its meaning?

Work effectively in multidisciplinary team, adapting to the particularities of each team and given roles	( )
Perform consulting, helping colleagues, other professionals, and the healthcare system	( )
Communicate effectively to promote understanding and resolve conflicts, aiming to ensure the success of teamwork	( )
Perform teamwork, aiming to ensure patient safety	( )
Communicate about ethical issues with other health professionals	( )
Demonstrate basic leadership skills	( )
Engage in the management of human and health care resources	( )
None of these Key Competencies	( )
